# A Hybrid Optimization from Two Virtual Physical Force Algorithms for Dynamic Node Deployment in WSN Applications

**DOI:** 10.3390/s19235108

**Published:** 2019-11-22

**Authors:** Qiang Li, Qiang Yi, Rongxin Tang, Xin Qian, Kai Yuan, Shiyun Liu

**Affiliations:** 1 Department of Physics, School of Science, Nanchang University, Nanchang 330031, China; 2 Institute of Space Science and Technology, Nanchang University, Nanchang 330031, China; 3 One Microsoft Way, Redmond, WA 98052-6399, USA; 4 Usun Microelectronics, Nanchang 330072, China

**Keywords:** wireless sensor networks, dusty plasma crystallization, virtual force algorithm, hybrid optimization

## Abstract

With the rapid development of unmanned aerial vehicle in space exploration and national defense, large-scale wireless sensor network (WSN) became an important and effective technology. It may require highly accurate locating for the nodes in some real applications. The dynamic node topology control of a large-scale WSN in an unmanned region becomes a hot research topic recently, which helps improve the system connectivity and coverage. In this paper, a hybrid optimization based on two different virtual force algorithms inspired by the interactions among physical sensor nodes is proposed to address the self-consistent node deployment in a large-scale WSN. At the early stage, the deployment algorithm was to deploy the sensor nodes by leveraging the particle motions in dusty plasma to achieve the hexagonal topology of the so-called “Yukawa crystal”. After that, another virtual exchange force model was combined to present a hybrid optimization, which could yield perfect hexagonal topology, better network uniformity, higher coverage rate, and faster convergence speed. The influence of node position, velocity, and acceleration during the node deployment stage on the final network topology are carefully discussed for this scheme. It can aid engineers to control the network topology for a large number of wireless sensors with affordable system cost by choosing suitable parameters based on physical environments or application scenarios in the near future.

## 1. Introduction

Wireless sensor network (WSN) is currently a hot research topic, which covers electronics, remote sensing, wireless communication, and other cutting-edge areas. The sensors can be deployed in an unmanned or harsh environment to detect the information of the target area due to the robustness and self-organization of the WSN. It has been widely used in national defense, environment monitoring, space exploration, traffic control, etc. [1,2,3]. As the rapid development of unmanned aerial vehicle in space exploration, the accurate topology control for a large number of wireless sensors would become an important issue in the near future [4,5].

For applications which require a massive number of sensor nodes, the sensor network should meet the performance requirements imposed by diverse applications and provide maximal coverage. To maximize the network quality and reduce the configuration cost, it has been proved that a hexagonal structure is the best topology in a two-dimensional network. It provides the maximum coverage area with the minimum number of sensor nodes and minimum energy consumption [6,7].

Currently, many scientists are paying great attention to the node deployment and system topology control in WSN applications based on the VFAs. Howard et al. proposed a dynamic deployment algorithm based on artificial potential field regarded mobile micro robots as sensor nodes [8]. Heo and Varshney improved the virtual force algorithm by adding certain constraints to the virtual force function [9]. Based on the theory of virtual potential field and disc packing, Chakrabarty and Zou used attractive and repulsive forces to determine the virtual motion paths and movement rate of sensor nodes. A probabilistic target localization algorithm was presented to enhance the field coverage [10]. To respond to sudden events or disasters quickly, Garetto et al. investigated a distributed relocation method with a regular tessellation of the geographical area [11]. Tang et al. implemented the algorithm computation and analyzed the details of influence of experimental parameters on Garetto’s algorithm [12]. Yu et al. introduced newly algorithms based on the virtual spring force [13] and the van der Waals force [14]. These two algorithms yield good uniformity, field coverage, and system convergence.

Moreover, similar to the traditional VFAs, Toumpis et al. showed a spatial distribution of wireless nodes in a traffic pattern that is identical to the electrostatic field by substituting the traffic sources with a distribution of positive electric charge and the traffic sinks by a distribution of negative charge [15]. Pac et al. proposed a novel approach inspired by compressible fluids as a distributed and scalable solution. In that work, they modeled the sensor network as a fluid body, and treated each sensor as a fluid element [16]. Tang et al. presented a self-consistent method of large-scale deployment in WSN applications by leveraging the phenomena that the dusty particles can automatically form Yukawa crystal structure (hexagonal topology). The influence of computation scale and shielding length on this algorithm were discussed in detail [17]. Especially, the VFA-DP can be easily extended to the z-axis in space, i.e., it has good potential to achieve a 3D network deployment.

However, it should be noted that most of the virtual force-based strategies only consider one kind of virtual force or physical model in a 2D plane. According to statistical analysis, the final network topology from one may have some twisted structure or coverage hole in the inner region due to different initial sensor network [18]. In addition, the advantages and disadvantages of different kinds of virtual or physical forces may differ, e.g., the computation complexity, system stability, etc. Those advantages and disadvantages could make significant impact on the efficiency of the deployment and final topology of the WSN. Based on our previous works on the VFA-DP and VFA-LJ [12,17], the VFA-DP concentrates more on a global distribution, and the VFA-LJ focuses on the local distribution. They use different physical forces and screening rules. Nevertheless, their distributed computation steps are almost equivalent. Therefore, our motivation for the present work is to merge their advantages in computation complexity and network topology. Some disadvantages from one physical virtual force can be fixed by the other physical force. The hybrid optimization for node deployment strategy is the following:

(1) A novel VFA scenario based on physical laws in a dusty plasma system (VFA-DP) was applied at the early stage of sensor network deployment [17]. It is derived from the studies of crystalline structure in condensed matter physics. The nodes can form hexagonal topology at the final stage. The central attracting force provides a screening effect via exponential decay [19,20]. The VFA-DP has good convergence rate with lower communication-related energy cost.

(2) A VFA based on virtual exchange force (Lennard–Jones Potential, the so-called VFA-LJ) was adopted to further improve the network topology [11,12]. The node positions from VFA-DP can be regarded as the initial deployment for the VFA-LJ. The corresponding velocity and the acceleration information were also used as the initial conditions at the first time step in the VFA-LJ calculation. The advantages of these two original physical force algorithms are merged.

(3) Detailed comparisons were proposed between the hybrid algorithm and the existing VFA-DP/VFA-LJ algorithms. The influence of velocity and acceleration parameters on this hybrid scheme was investigated. This optimization yields perfect hexagonal topology, better network uniformity, and higher coverage rate. In addition, it makes the equilibrium distance between any two nodes more uniform.

Since the real applications of large-scale WSN are related to the system and the environment, how to obtain a balance between total deployment time and network performance is a key issue. This hybrid scheme aims at effectively reducing the computation complexity by selecting a suitable transition time from VFA-DP to VFA-LJ. The investigation on the node velocity and acceleration during the deployment process can also provide the valuable guidance to the engineers to choose suitable algorithm parameters to deploy a large number of wireless sensors in different physical environment or application scenarios. It also indicates that the 3D deployment of a WSN may be optimized by combining different VFAs in the near future.

The rest of this paper is organized as follows. Section 2 proposes the combination of two virtual physical force algorithms inspired by the dusty plasma crystallization and the Lennard–Jones potential to present a hybrid optimization scenario in the WSN’s topology control. The methodology of performance evaluation is explained in detail in Section 3. The simulation results of different time scale and the influence of node parameters during the deploying process are presented in Section 4. Finally, the conclusion and directions for future work are summarized in Section 5.

## 2. Combination of Virtual Force Algorithms

**Figure 1:**
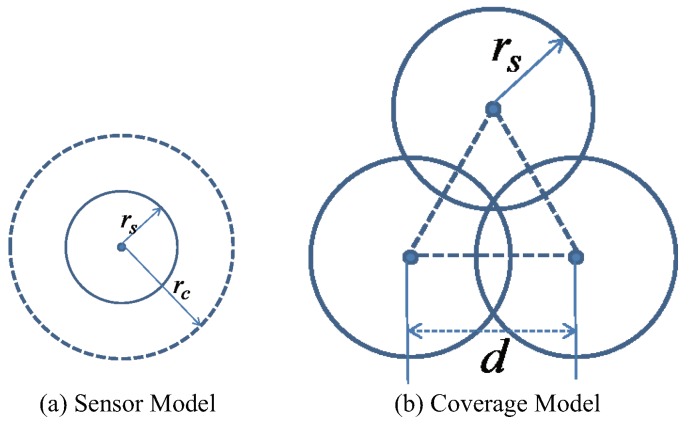
Both the sensing and communication ranges were assumed to be a disk, with radii of $r_{s}$ and $r_{c}$, respectively: (**a**) sensor model; and (**b**) coverage model.

For this study, we assumed that all sensor nodes had identical capabilities in sensing, communication, computation, and mobility. The targeted application field consists of a 2D plane, and sensors can move freely within the plane. Each sensor node has a sensing radius of $r_{s}$ and a communication radius of $r_{c}$. As illustrated in Figure 1, both the sensing and communication ranges are assumed to be a disk. Within the sensing radius, the sensor can detect the local environment. Within the communication range, the sensor node can communicate with any other sensor falling into the disk area. Every node has the ability to know its own location and can exchange its position (even velocity or acceleration if the application needs) with sensors over the communication range. Details of the communications between the sensors are not discussed here.

We then set the sensing radius $r_{s}=1$, which is a normalized radius depending on different wireless sensor. If the real sensing radius of one sensor node is greater, the final coverage of the whole wireless sensor network is larger. The communication radius $r_{c}$ is also normalized. Generally, the communication range of one sensor is limited. However, it is necessary to ensure that the adjacent sensor nodes can communicate with each other. Based on the topology of a perfect hexagonal network, the distance between two adjacent nodes is $\sqrt{3}r_{s}$ (see Figure 1b). $r_{c}$ must be larger than $\sqrt{3}r_{s}$. Furthermore, the communication radius is also used to shield the nodes with a longer distance in virtual force algorithm. Only the nodes inside the $r_{c}$ will be involved in these two VFA algorithms.

Our strategy gives a concept of node deployment solution for a large scale WSN. The sensor communication will affect the performance and reduce the effect of the final hexagonal topology. After one sensor approaches its estimated position, it also should wait for a while to receive the updating information from neighbor nodes. This may cause some time delay for the whole process. Besides, to avoid the possible coverage hole from sensor’s location error, it can be solved by slightly decreasing the fixed distance $\sqrt{3}r_{s}$ (for a perfect hexagonal topology). However, this can reduce the whole coverage area and cause some redundancy.

The theoretical models of two adopted VFAs are introduced in the present section, and their advantages and disadvantages are presented. The combination scenario in WSN applications is developed based on the two VFAs.

### 2.1. Virtual Force Algorithm Based on Dusty Plasma Crystallization

In the VFA based on dusty plasma crystallization, each wireless sensor is treated as a dust particle. The algorithm finds a final deployment distribution of hexagonal topology. The nonlinear equation of motion [17,19] for a dust particle *i* of the mass *m* and the charge *Q* is given by

(1) $$m\frac{d^{2}{\vec{r}}_{i}}{dt^{2}}=-\nabla \phi_{i}-m\upsilon \frac{d{\vec{r}}_{i}}{dt},$$

where the electrostatic potential $\phi_{i}$ is

(2) $$\phi_{i}=k{\vec{r}}_{i}^{2}\left(t\right)+\sum_{i\neq j}^{N}\frac{Q^{2}}{4\pi \varepsilon_{0}r_{ij}}e^{-\frac{r_{ij}}{\lambda_{D}}}.$$

Here, ${\vec{r}}_{i}$ is the radial distance from the center, $r_{ij}$ is the distance between two particles *i* and *j*, υ is the damping coefficient, $\lambda_{D}$ is the Debye length of the plasma, and the sum is calculated over all of the particles within the communication range. As for Equation (2), the first term on the right-hand side is the externally imposed harmonic potential that provides radial confinement, whereas the spring constant *k* can control inter-particle separation. The second term represents the Yukawa potential that exponentially decreases once $r_{ij}$ exceeds $\lambda_{D}$.

A second-order leap-frog scheme was adopted in the present simulations to solve Equation (1) numerically. Mathematically, the scheme is given by the following formulas:

(3) $${\vec{r}}_{k+1}={\vec{r}}_{k}+{\vec{v}}_{k}dt+\frac{1}{2}{\vec{a}}_{k}dt^{2}$$

(4) $${\vec{v}}_{k+1}={\vec{v}}_{k}+\frac{1}{2}\left({\vec{a}}_{k}+{\vec{a}}_{k+1}\right)dt$$

where *v* and *a* are the particle velocity and acceleration at each time step, respectively.

This algorithm yields better field coverage with perfect hexagonal topology and good system uniformity, even in 3D space [19]. It can be considered as an aid to fast deployment experiments while the WSN is formed by thousands of wireless sensors in a large-scale area. In the VFA-DP, the shielding length is defined by the parameter $\lambda_{D}$. When $\lambda_{D}$ = 0.0526, the VFA-DP calculates the total virtual Yukawa potential from the closest adjacent nodes of a given sensor. However, in such a case, the final equilibrium distance between two nodes cannot be the same from the center to the edge (we discuss this in Section 4). If the user wants to obtain better equilibrium distances, a larger $\lambda_{D}$ is required and more sensor nodes are involved to calculate the total virtual force [17]. It increases the computation time of the VFA-DP. Thus, for some applications, the Yukawa potential from enough nodes needs to be considered in the algorithm, which would lead to the dramatic enhancement of the computation complexity.

### 2.2. Virtual Force Algorithm Based on Lennard–Jones Potential

Another concerned VFA model was developed based on a second-order differential equation of node motions [11,12]. In this algorithm, the motion of nodes are varying, and consequently achieve a dynamic equilibrium in a physical system. The equation of a given node *i* is

(5) $$m\frac{d^{2}r_{i}\left(t\right)}{dt^{2}}=F_{i}\left(t\right)=F_{i}^{e}\left(t\right)+F_{i}^{f}\left(t\right)$$

where *m* is the virtual mass of each node, $F_{i}^{e}\left(t\right)$ is the total exchange force emerged from other nodes, and $F_{i}^{f}\left(t\right)$ is the friction force, which reduces the node velocity to achieve the equilibrium state.

Once the distance $d_{ik}\left(t\right)$ between two given nodes *i* and *k* at time *t* is less than the communication radius $R_{c}$, the exchange force between these two nodes would be activated. A(i,t) is the set of nodes which contribute to exchange forces acting on node *i* at time *t*. Then, k∈A(i,t). The formula of total exchange forces $F_{i}^{e}\left(t\right)$ on node *i* at time *t* is defined as

(6) $$F_{i}^{e}\left(t\right)=\sum_{k\in A(i,t)}G\frac{\left(d_{ik}\left(t\right)-D_{m}\right)}{d_{ik}\left(t\right)\left(R_{c}-d_{ik}\left(t\right)\right)}{\hat{r}}_{ik}\left(t\right)$$

where ${\hat{r}}_{ik}\left(t\right)$ is the normalized vector of $r_{ik}\left(t\right)$, $D_{m}$ is the equilibrium distance, and *G* is a constant which significantly affects the node speed.

The friction forces $F_{i}^{f}\left(t\right)$ acting on node *i* at time *t* can be written as

(7) $$F_{i}^{f}\left(t\right)=\left\{\begin{array}{c} -\min\left(k_{0}{\hat{F}}_{i}^{e}\left(t\right)\;F_{i}^{e}\left(t\right)\right),\;\;\mathrm{if}\;v_{i}\left(t\right)=0\\ -k_{\upsilon }v_{i}\left(t\right),\;\;\mathrm{if}\;v_{i}\left(t\right)\neq 0 \end{array}\right.,$$

where ${\hat{F}}_{i}^{e}\left(t\right)$ is the normalized vector of $F_{i}^{e}\left(t\right)$, $v_{i}\left(t\right)$ is the velocity of node *i*, $k_{0}$ is the static friction factor, and $k_{\upsilon }$ is the viscous friction factor. While $v_{i}\left(t\right)=0$, the static Coulomb friction $-k_{0}{\hat{F}}_{i}^{e}\left(t\right)$ suppresses the movement of node *i*. Once $v_{i}\left(t\right)\neq 0$, the viscous friction $-k_{\upsilon }v_{i}\left(t\right)$ decelerates the node *i*.

In the VFA-LJ, the shielding length is $\sqrt{3}r_{s}<r_{c}<3r_{s}$, which means it only concerns the direct force from the closest adjacent nodes of a given sensor. Six nodes, at most, can simultaneously impact this given node. Therefore, the computation time is faster. The VFA-LJ reduces the computation complexity, which effectively yields the reduction of computation time and energy consumption in the process of node deployment. It is appropriated to be utilized in a small-scale WSN. However, if a larger shielding length were used, the final network topology would be worse [12].

### 2.3. Hybrid Optimization Based on Above Two VFAs

Since the basic distributed algorithm of these two VFAs are similar, the detailed process of the hybrid optimization are as follows:

(1) Initially, all sensor nodes were randomly scattered in a circular area.

(2) By setting the shielding length, the sum of virtual physical force for node *i* will be generated between selected neighboring nodes. The VFA-DP algorithm is employed first to form a good hexagonal topology for the sensor network in a large area.

(3) The pseudo-code of the leapfrog schema solutions for the VFA-DP and VFA-LJ is shown in Algorithm 1. Perform the following steps for all nodes in a loop.

(4) Repeat Step (3), and update the coordinate, speed, and acceleration information of each node every dt time until the whole network is relatively stable or approaches the transition time.

(5) All the relevant parameters at a suitable time step are saved. The node positions, velocity, and accelerations are normalized based on the average node distance of the whole network.

(6) All the saved parameters are transferred to the VFA-LJ. The final node positions determined by VFA-DP method is regarded as initial deployment for the VFA-LJ algorithm. The corresponding node velocity and the acceleration can also be utilized as the initial conditions for the VFA-LJ calculation if applications require.

(7) Use the VFA-LJ algorithm to achieve the final optimized network topology. The pseudo code is the same as Algorithm 1.
**Algorithm 1** Distributed algorithms in VFA-DP and VFA-LJ in wireless sensor networks.
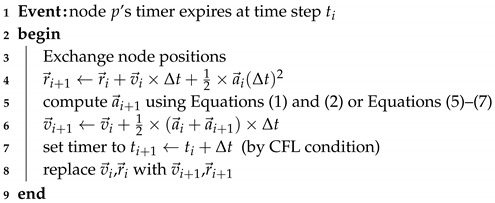



In this paper, we used a computer with i7-6700@3.4 GHz CPU and 8 G memory and ran our code in Microsoft Visual C++ 2012 to achieve the virtual force calculation. The corresponding time step in this simulation tools was 0.08 s.

## 3. Performance Evaluation

In the present section, a novel performance metric called the pair-correlation diversion (PCD) is introduced to quantitatively analyze the proximity from any network topology to a perfect hexagon configuration [12,17]. It is based on the pair-correlation function g(r)=(N(r,Δ)S)/(2πrΔN) in the crystal structure, which denotes the probability of finding two nodes separated by a distance *r*. *S* is the area occupied by a 2D system. *N* is the number of nodes contained in *S*, and N(r,Δ) indicates the number of nodes located between r−Δ/2 and r+Δ/2.

(8) $$\delta \left(\Omega ,\Omega_{H}\right)=\frac{\int_{0}^{r_{T}}{\left\|g_{\Omega }\left(r\right)-g_{\Omega_{H}\left(r\right)}\right\|}^{2}dr}{\int_{0}^{r_{T}}{\left\|g_{\Omega_{H}\left(r\right)}\right\|}^{2}dr}\;,$$

where Ω is the network topology to be analyzed, $\Omega_{H}$ is the perfect hexagonal topology, and $r_{T}$ is the bound of the radial distance [11].

This metric indicates the uniformity of the sensor network. Moreover, it can be utilized to characterize the convergence rate of any virtual force algorithm. The slope of the pair correlation function curve, as a function of the simulation time steps, indicates how fast the virtual force algorithm converges. For a WSN, when the value of $\delta (\Omega ,\Omega_{H})$ is small, the network coverage topology approximates a hexagonal structure. In addition, the convergence rate can be obtained according to the decrease of the PCD curve. When a network reaches its steady state, the PCD value should also become stable.

Besides the PCD, another widely-used metric is adopted to evaluate the uniformity of the final node distribution. The average local standard deviation *U* of the distances between sensor nodes [9,11] is defined as

(9) $$U=\frac{1}{N}\sum_{i=1}^{N}U_{i}$$

(10) $$U_{i}={\left(\frac{1}{K_{i}}\sum_{j=1}^{K_{i}}{\left(D_{ij}-M_{i}\right)}^{2}\right)}^{\frac{1}{2}},$$

where *N* is the total number of nodes, $K_{i}$ is the number of neighbors for the *i*th node, $D_{ij}$ is the distance between the *i*th and *j*th nodes, and $M_{i}$ is the mean of intermodal distance between the *i*th node and its neighbors. The method helps to further test the validation and the effect of these algorithms.

## 4. Simulations and Discussions

In this section, the detailed simulation results using the hybrid optimization are presented in order to compare it with VFA-DP [17] and VFA-LJ [11]. The comparisons mainly include three aspects: (i) the similarity between the resulting deployment topology and a perfect hexagon; (ii) the convergence rate toward a stable hexagonal structure; and (iii) the influence of velocity and acceleration parameters on this hybrid optimization method.

The number of sensor nodes was set to be N = 1500 in the whole target region. In the VFA-DP, the initial parameters were chosen based on the fundamental experiments by Ma and Bhattacharjee [19]. In particular, to match the purpose of present study, the parameters were set as Q = 15,700 e, m = 5.2 × 10^−13^ kg, υ = 2.7 s^−1^, k = 2.4 × 10^−13^ kg/s^2^, and $\lambda_{D}$ = 0.0526 µm. Once the calculations based on the VFA-DP were completed, the node positions, velocities, and accelerations were normalized and then transferred to the VFA-LJ. The normalization was valid and necessary since the present strategy concentrated on the concept of node deployment solution for a large-scale WSN, thus the sensor communication and data transmission were not considered. Therefore, in the VFA-LJ, the virtual mass was normalized to m=1 since each sensor was treated as a particle without considering the hardware design and its mechanical parts. The sensing radius, which significantly relies on the wireless sensor itself, was normalized to $R_{s}=1$. It could be expected that the final coverage of the whole WSN increases with the real sensing radius of a single node. Then, based on the previous work [12], the initial parameters for the VFA-LJ were set to be G=0.1, $k_{0}=1.0$ e^−5^ and $k_{\upsilon }=0.1$.

**Figure 2:**
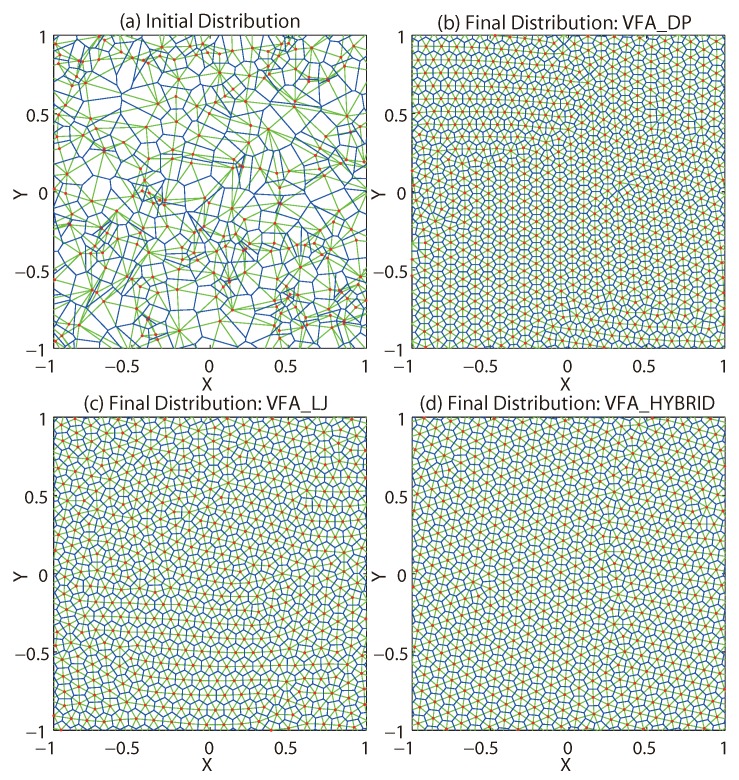
Network topology located within the central square area: (**a**) initial node distribution; (**b**) final node distribution from VFA-DP algorithm; (**c**) final node distribution from VFA-LJ algorithm; and (**d**) final node distribution from VFA-Hybrid algorithm.

The network topologies of the WSN located within the central square of a target area are shown in Figure 2. All the sensor nodes were randomly deployed at the initial stage (Figure 2a). Three algorithms were utilized separately to obtain the final network topology. Figure 2b–d shows the final node distributions from VFA-DP, VFA-LJ, and VFA-Hybrid algorithms, respectively. It is obvious that both VFA-DP and VFA-Hybrid yield better hexagonal structure than that of VFA-LJ since the fundamental VFA inspired from dusty plasma crystallization can form the topology which is nearly hexagonal in a physical system [17,19]. The dusty plasma system has a global attractive force, which is related to the pressure in the actual physical world. This pressure helps the matter transform into liquid or solid crystal. Therefore, it is no wonder that the dusty plasma results in a better lattice and faster convergence.

**Figure 3:**
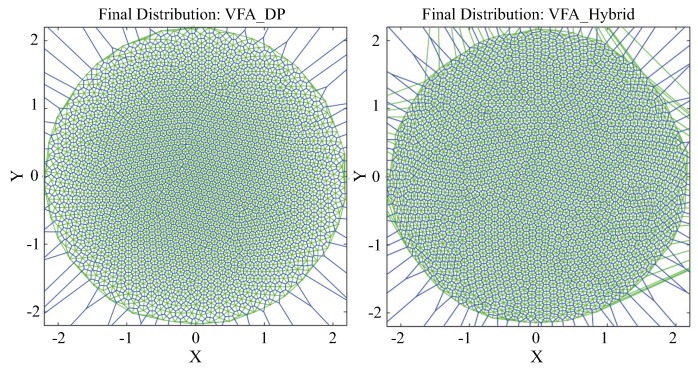
The global view of the final distributions from the VFA-DP and VFA-Hybrid algorithms.

However, in the final structure yielded by VFA-DP, the equilibrium distances between every two nodes around the corner are greater than those in the center of the square. Figure 3 shows the global view of the final distributions from VFA-DP and VFA-Hybrid. It is very clear that the edge of the target circles from VFA-DP and VFA-Hybrid are different. The sensors around the edge of the final distribution from VFA-DP (Figure 3, left) are much sparser. However, the central region is much tighter. Finally, the VFA-Hybrid improved these global effects. The average equilibrium distance obtained from both two algorithms for the specified radius (where sensor locates) are listed in Table 1. It also clearly shows that the central region has been compressed, which reduces the whole coverage area and causes some redundancy. Furthermore, the VFA-LJ process significantly improves the uniformity of the whole network system while the equilibrium distance from the VFA-Hybrid algorithm is more equivalent.

**Figure 4:**
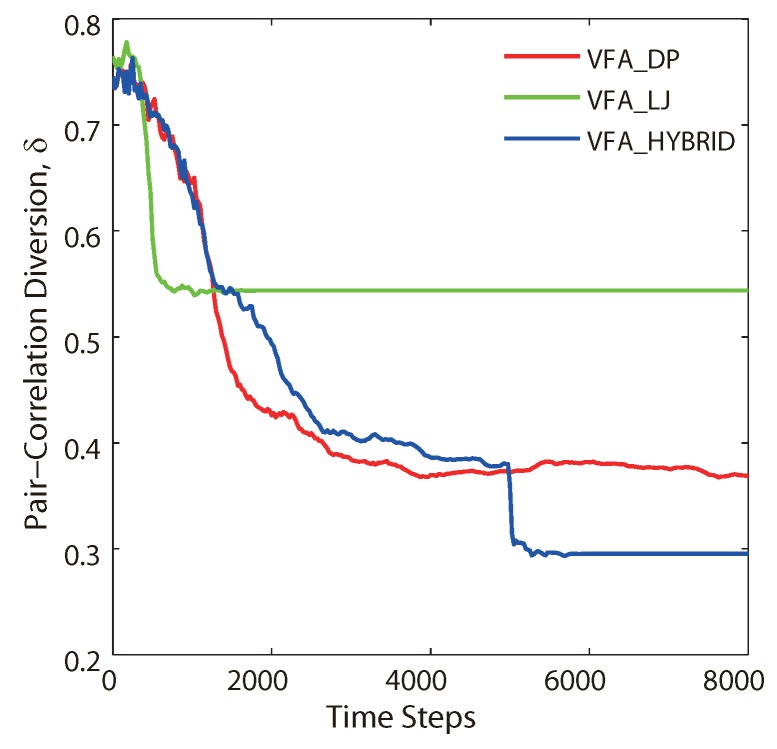
Performance evaluation profiles of the PCD $\delta (\Omega ,\Omega_{H})$ as a function of the time step from VFA-LJ, VFA-DP and VFA-Hybrid algorithms, respectively.

Figure 4 illustrates the performance evaluation profiles of the pair correlation diversion $\delta (\Omega ,\Omega_{H})$ as a function of the time steps of the VFA-DP, VFA-LJ, and VFA-Hybrid algorithms, respectively, which correspond to the final distributions in Figure 2b–d. The lower PCD value indicates better hexagonal network topology. Based on the simulation results, the converged diversion value for the original VFA-LJ is approximately 0.55 after 800 time steps. On the other hand, the diversion value for the original VFA-DP is approximately 0.4 after 2100 time steps. It indicates that VFA-DP achieves better hexagonal network topology, which is consistent with Figure 2b. For VFA-Hybrid, the involved virtual force switched from Yukawa potential (Equations (1) and (2)) to Lennard–Jones potential (Equations (5)–(7)) at the 5000th time step without considering the node velocity and the acceleration. After the algorithm switching, VFA-Hybrid further improved the network structure and the corresponding PCD value is 0.3. As shown in Figure 2d, the VFA-Hybrid algorithm had best hexagonal distribution.

**Figure 5:**
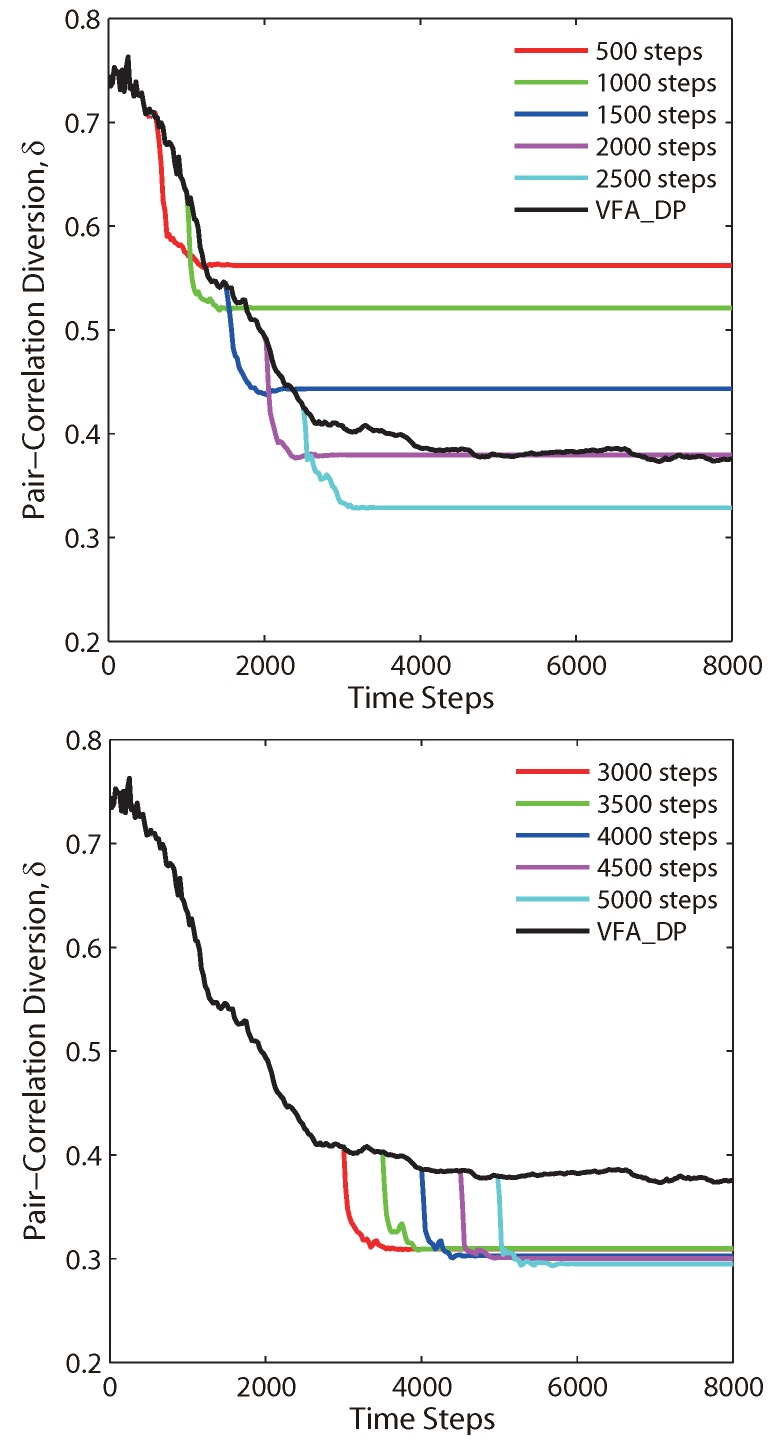
The PCD $\delta (\Omega ,\Omega_{H})$ of VFA-Hybrid algorithm as a function of the time step for the specified switch time transferred from VFA-DP to VFA-LJ.

The whole process of VFA-Hybrid can be summarized into two steps. For the first step, the VFA-DP algorithm deploys the WSN into a hexagonal topology from the initial random deployment. For the second step, the virtual force based on the Lennard–Jones potential further improves the sensor network structure and eventually yields a better topology. On the other hand, the effect of VFA-Hybrid is impacted by the time of switch. Such impacts are shown in Figure 5. If the hybrid algorithm switches at earlier time steps, e.g., time steps 500–2000, the VFA-hybrid would yield a quasi-hexagonal network topology, but it is still not good enough. The reason is that the hexagonal structure is still being improved by VFA-DP, while the corresponding PCD value is still deceasing. If the hybrid algorithm switches at later time steps (2500–5000 time steps), the structure driven by VFA-DP almost reaches an equilibrium state and forms a hexagonal network (the corresponding PCD values were close to 0.4). Therefore, VFA-Hybrid further improves this network distribution into a better hexagonal topology, whose PCD values decreased to 0.3. The results indicate that engineers can choose an appropriate switch time in the VFA-Hybrid, depending on the different environments or actual parameters, to reduce the computation complexity and save the energy consumption for all sensor nodes. It is very useful in physical large scale WSN applications.

**Figure 6:**
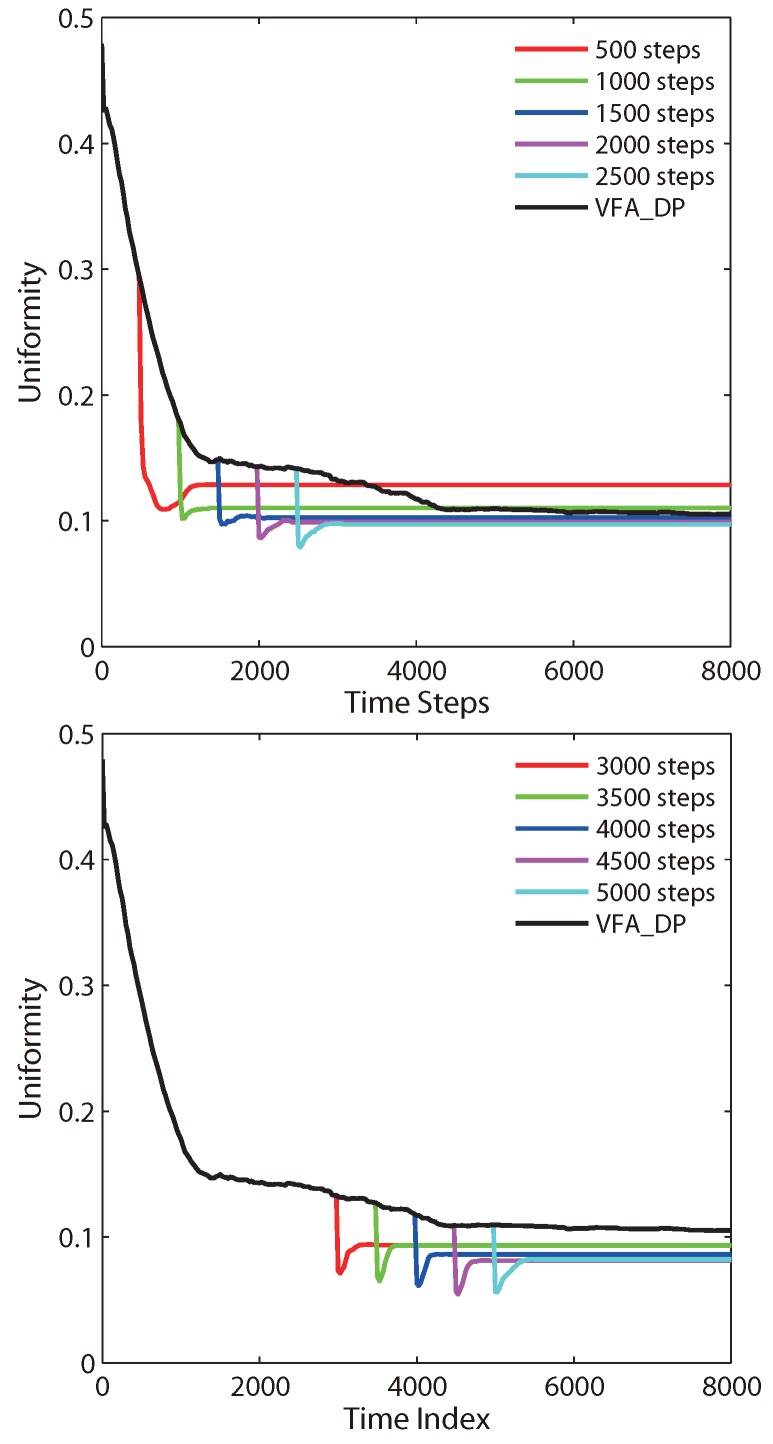
Typical profiles of system uniformity, as a function of the time step, calculated for the specified switch time from VFA-DP to VFA-LJ.

Figure 6 shows the typical profiles of system uniformity for VFA-DP and VFA-Hybrid, as a function of time step obtained from Equations (8) and (9) for the specified switch time. Obviously, the uniformity of the whole network is further improved and the lower *U*-values are obtained when the virtual force of Lennard–Jones potential is adopted. It is also consistent with the results shown in Table 1 and Figure 2.

It should be noticed that both VFA-DP (Equation (1)) and VFA-LJ (Equation (5)) need to calculate the node velocity and the acceleration at each time step. Furthermore, to check their influence on the consequent effect of the VFA-hybrid algorithm, the velocity and the acceleration information of each sensor node are considered as additional initial conditions when the virtual force calculation transferred from VFA-DP to VFA-LJ.

**Figure 7:**
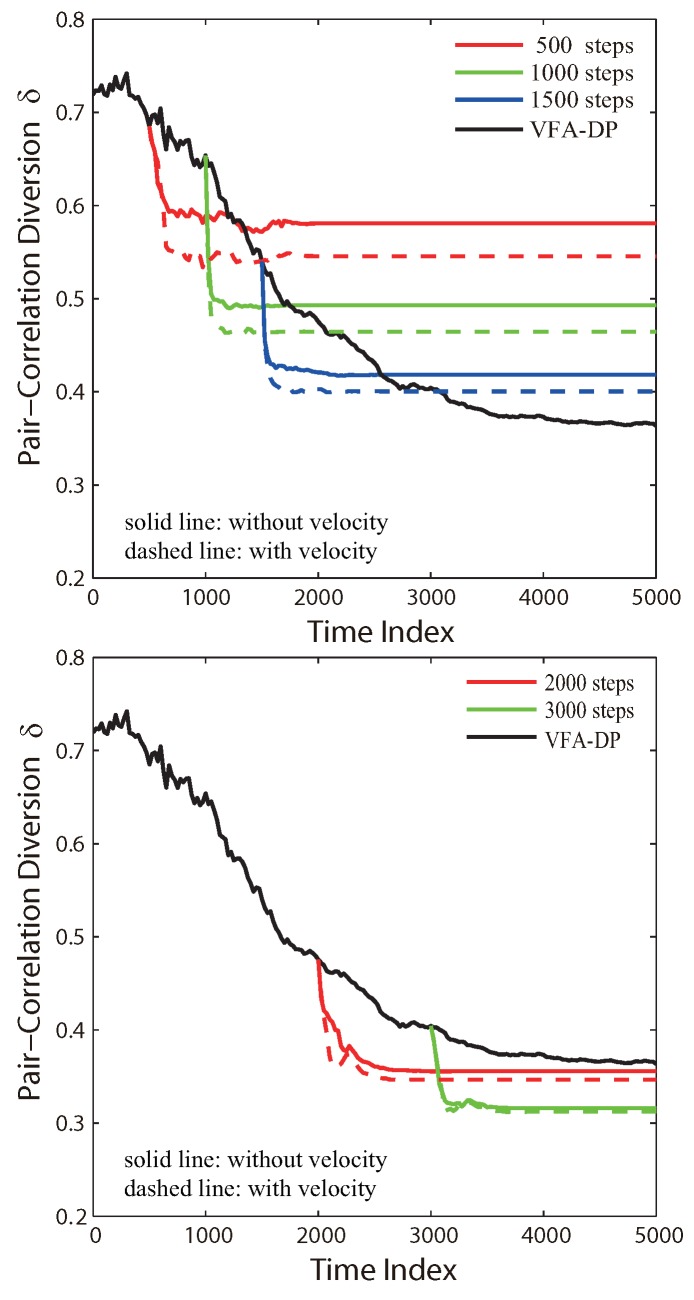
The PCD $\delta (\Omega ,\Omega_{H})$ of VFA_Hybrid algorithm as a function of the time step, for the specified switching time, by taking into account the influence of node velocity when hybrid optimization transferred from VFA_DP to VFA_LJ.

Figure 7 illustrates the PCD profiles for the VFA-Hybrid algorithm as a function of time step. The node velocity was treated as an additional initial condition in the hybrid optimization. For the different switch time, the hybrid algorithm with the node velocity brought from VFA-DP can further improve the network distribution to achieve better hexagonal structure. The reason is implied in Figure 7. The dashed profiles (with velocity information) yield lower PCD values than those of the solid profiles (without velocity information). When the algorithms switch at early time steps, the node velocity remarkably influences the network improvement since the whole sensor system has not yet approached the equilibrium state. Each node is still moving quickly toward its next position determined by the virtual force algorithm. On the contrary, if the hybrid optimization chooses a later switch time, the network distribution yielded by VFA-DP is almost an equilibrium state. Sensors only slightly move around their fixed positions, which results in small velocity, i.e., the velocity no longer makes significant contribution to the hybrid algorithm.

**Figure 8:**
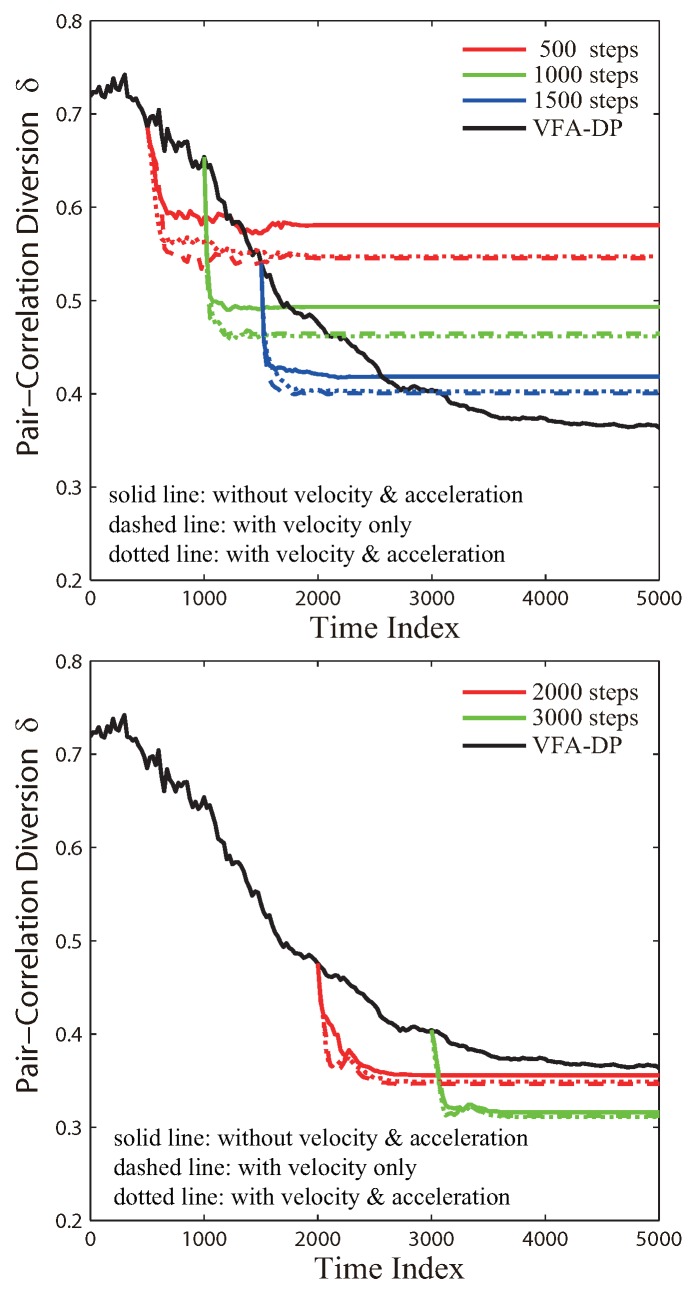
As specified in Figure 7, except taking into account the node acceleration in simulations.

Similarly, Figure 8 shows the PCD profiles for the VFA-Hybrid algorithm by taking both the velocity and the acceleration into account. According to Figure 7, the dotted profiles (with acceleration) are very close to the dashed profiles (without acceleration). It indicates that the acceleration of sensor nodes does not significantly improve the network distribution. The reason is that the acceleration derived from the second-order motion equation is small. As a result, the node velocity plays a more important role in the hybrid algorithm. In summary, the node velocity is a very important factor that needs to be taken into account by engineers who require high quality and high accuracy for the network topology in realistic WSN applications.

Furthermore, the convergence rate and the computation time of the three algorithms are appropriate indices to evaluate the algorithm complexity and the system energy cost. The algorithm runtime calculated from the two parts of the VFA-Hybrid algorithm for the specified time steps are listed in Table 2, corresponding to the experiments illustrated in Figure 4. Generally, in VFA-Hybrid, VFA-DP contributes more than VFA-LJ on the computation time consumption. The reason is that the dusty plasma crystallization always considers a large number of sensor nodes in Equations (1) and (2), which leads to more computation complexity. Thus, if the algorithm switch is postponed, VFA-DP requires more system cost and time. Nevertheless, it achieves better network topology (lower PCD value) as a fundamental structure. The following part (VFA-LJ) then spends less system cost to obtain a perfect hexagonal network. It could be concluded that the hybrid algorithm combines the advantages of VFA-DP and VFA-LJ, which afford a greater energy availability based on suitable parameters.

## 5. Conclusions

In this paper, a hybrid optimization is presented for sensor node deployment strategy in a large-scale WSN application. It was developed based on the combination of two previous algorithms, which are the algorithm inspired by the dusty plasma crystallization (VFA-DP) and the algorithm based on the Lennard–Jones Potential (VFA-LJ). The advantages of two original virtual force algorithms are merged. This hybrid optimization can yield perfect hexagonal topology, better network uniformity and higher coverage rate. It also makes the equilibrium distance between two nodes more uniform.

It can be noted that the optimized method is a theoretical analysis which provides a hexagonal sensor network with the maximum coverage area and the minimum number of sensor nodes in the target area. The influence of node velocity, acceleration and the transition time on the proposed hybrid algorithm were carefully analyzed. Comparing with the node acceleration, the node velocity makes more contribution to the final effects of the hybrid algorithm and needs to be taken into account by the engineers who require high quality and high accuracy for the WSN applications.

To summarize, by choosing suitable physical forces, the hybrid optimization can merge the advantages of these physical force calculations, reduce the computation complexity, and save the system energy consumption. Some disadvantages from one physical virtual force can be fixed by the other physical virtual force. The engineers may not consider detailed parameter optimizations to improve the final networks from one virtual force. In our next plan, more different kinds of physical virtual forces will be discussed to improve the system flexibility. On the other hand, the 3D optimization via some physical virtual forces with their advantages is also an interesting topic. It can help engineers to effectively deploy a large number of wireless sensors in the near future.

## Figures and Tables

**Table 1:** The average equilibrium distance between two sensor nodes calculated from both VFA-DP and VFA-Hybrid algorithms for the specified radius where the sensor is located.

Radius	[0, 0.5)	[0.5, 1.1)	[1.1, 1.6)	[1.6, 2.2)
VFA-DP	0.093752	0.108681	0.118154	0.129182
VFA-Hybrid	0.110189	0.109857	0.112812	0.110152

**Table 2:** Algorithm runtime and PCD values calculated from two parts of VFA-Hybrid algorithm for the specified time steps corresponding to Figure 4.

First Part: VFA-DP			Second Part: VFA-LJ			VFA-Hybrid
Steps	Time (ms)	PCD	Steps	Time (ms)	PCD	T_Total (ms)
500	422,857	0.7088	2025	752,726	0.6427	1,175,583
1000	858,600	0.6329	1050	440,052	0.5355	1,298,652
1500	1,505,266	0.5450	1100	400,764	0.4569	1,906,030
2000	2,204,272	0.4936	775	319,501	0.4114	2,523,773
2500	2,844,781	0.4254	825	337,194	0.3372	3,181,975
3000	3,423,264	0.4077	800	336,613	0.3184	3,759,877
3500	3,999,622	0.4032	825	378,600	0.3081	4,378,222
4000	4,566,203	0.3862	750	231,815	0.3051	4,798,018
4500	5,043,091	0.3846	800	266,133	0.3011	5,309,224
5000	5,518,119	0.3729	700	311,474	0.2838	5,829,593
